# Isolation and Identification of Anti-Inflammatory Peptide from Goose Blood Hydrolysate to Ameliorate LPS-Mediated Inflammation and Oxidative Stress in RAW264.7 Macrophages

**DOI:** 10.3390/molecules27248816

**Published:** 2022-12-12

**Authors:** Yeye Du, Shuangjie Zhu, Ran Wang, Xingyong Chen, Kezhou Cai

**Affiliations:** 1 Engineering Research Center of Bio-Process, Hefei University of Technology, Ministry of Education, Hefei 230009, China; 2 School of Biological and Food Engineering, Chuzhou University, Chuzhou 239001, China; 3 College of Animal Science and Technology, Anhui Agricultural University, Hefei 230036, China

**Keywords:** goose blood, bioactive peptides, anti-inflammatory, digestive stability

## Abstract

This study was designed to isolate an anti-inflammatory activity oligopeptide from goose blood (GBP) for ameliorating LPS-mediated inflammation response and oxidative stress in RAW264.7 macrophages. In this study, GBP was isolated by tangential flow ultrafiltration system (TFUS) combined with size exclusion chromatography (SEC), ion exchange chromatography (IEC), and reversed-phase liquid chromatography (RP-LC), and then identified by liquid chromatography mass spectrometry (LC–MS/MS). The experiment results indicated that the amino acid sequence of oligopeptide with the best anti-inflammatory activity was IIe-Val-Tyr-Pro-Trp-Thr-Gln-Arg (IVYPWTQR), which had a molecular weight of 1062.5720 Da, and was derived from haemoglobin subunit beta OS in goose blood. In addition, IVYPWTQR was confirmed to have satisfactory stability and maintained high anti-inflammatory activity in a simulated gastrointestinal digestion. The mechanism by which the IVYPWTQR protected against LPS-mediated inflammation response was attributed to downregulating the *TLR4/NF-kB/iNOS* pathway. Moreover, IVYPWTQR ameliorated oxidative stress damage in inflammatory state was attributed to activating antioxidant defence system, which was regulated by *Keap-1*/*NRF2/HO-1* signalling pathway for decreasing the accumulation of reactive oxide species (ROS). In summary, these results indicated GBP could serve as a potential functional factor for prevention and improvement of inflammation mediated by LPS and provided an affordable dietary intervention strategy to prevent inflammation.

## 1. Introduction

Inflammation is the adaptive response to toxic stimuli and exogenous antigens, which play an important role in many diseases. Inflammation is accompanied by the occurrence of disease, can exacerbate the development of diseases, and can even lead to malignant tumours [1]. LPS-mediated inflammation is one of the crucial causes of chronic nonviral hepatitis caused by inappropriate diet, which can gradually deteriorate into liver cirrhosis, liver fibrosis, and liver cancer [2]. According to the epidemiological investigation report, LPS-mediated inflammation is one of the major diseases leading to morbidity and mortality worldwide [3,4]. At present, antibiotics are commonly used to alleviate nonviral hepatitis, but the abuse of antibiotics has strong side effects [5]. Due to the lack of specific therapeutic drugs for managing nonviral hepatitis, the treatment of nonviral hepatitis remains a major health problem worldwide.

Previous studies have confirmed that inappropriate diets (alcohol consumption, high-fat, high-cholesterol, and high-fructose diets) impair intestinal barrier function and accelerate the translocation of enterogenous lipopolysaccharide (LPS), which activate downstream pathways and the release of relevant inflammatory factors that lead to hepatitis [6,7]. Macrophages, especially Kupffer cells, may contribute to the development of inflammation by generating various inflammatory mediators (ROS and NO) and a series of inflammatory cytokines after being stimulated by LPS, such as IL-1β, IL-6 and TNF-α [8]. Furthermore, ROS may induce oxidative stress in nonviral hepatitis, which mediates apoptosis and inflammation and aggravates disease progression [9,10]. Therefore, inhibiting oxidative stress and the inflammatory cascade are effective strategies to ameliorate LPS-induced nonviral hepatitis.

In recent years, dietary bioactive peptides have been commonly used to ameliorate the inflammatory cascade and prevent disease aggravation in the liver due to their characteristics of being absorbable and safe [11,12]. Dietary bioactive peptides, such as tricholoma matsutake singer peptides [13], chicken liver peptides [14], marine collagen peptides [15], kefir peptides, [16] and Jinhua ham oligopeptides [7], have been shown to ameliorate liver damage. Although great efforts had been made, preventing inappropriate diet-induced inflammation remains a challenge.

Animal by-products are commonly regarded as a low-cost source of protein for the preparation of bioactive peptides by hydrolysation [17]. In China, approximately 800 million geese are bred annually, accounting for 40% of the world’s total, and a large amount of goose blood is produced as a by-product, which is regarded as fertilizer or animal feed [18]. Therefore, it is feasible and meaningful to use goose blood as a protein source to produce bioactive peptides with anti-inflammatory activity through a specific enzymatic hydrolysis process.

This study was designed to isolate an anti-inflammatory activity oligopeptide from goose blood (GBP) for ameliorating LPS-mediated inflammation and for a preliminary elucidation of the mechanisms by which target peptides prevent the LPS-mediated inflammatory cascade. This study may provide a new way for high-value processing and utilization of goose blood by-products. Simultaneously, it provided an economical and feasible strategy to prevent or mitigate chronic liver inflammation caused by inappropriate diet through dietary supplement of functional factors.

## 2. Results and Discussion

### 2.1. GBP Ameliorated LPS-Mediated Inflammation in Macrophages

**Figure 1:**
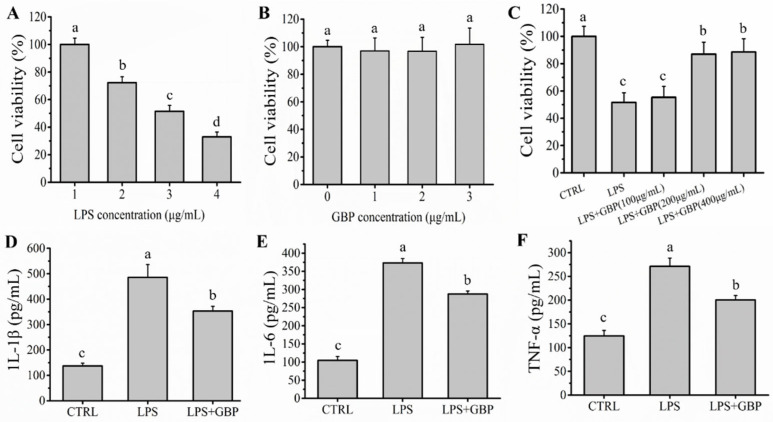
(**A**) The viability of RAW264.7 macrophages with different concentrations of LPS treatment; (**B**) The viability of RAW264.7 macrophages with different concentrations of GBP treatment; (**C**) Effects of the different concentrations of GBP pre-treatment on LPS-mediated RAW264.7 macrophages viability (LPS concentration: 3.0 μg/mL, GBP concentration: 100, 200 and 400 μg/mL); (**D**–**F**) Effects of the GBP pre-treatment on the levels of IL-1β, IL-6 and TNF-α on model of LPS-Mediated RAW264.7 macrophages damage. Different letters (a, b, c and d) in the figure represent significant differences between groups (*p* < 0.05).

The cytotoxicity of LPS and GBP to RAW264.7 macrophages was investigated to determine the concentration of LPS and GBP for use in further experiments. With increasing LPS treatment concentrations, the viability of RAW264.7 macrophages decreased significantly (*p* < 0.05) (Figure 1A). The viability of RAW264.7 macrophages was approximately 51.51 ± 4.29% in the presence of 3.0 μg/mL LPS, therefore, the LPS concentration resulting in 50% RAW264.7 macrophages death was around 3.0 μg/mL. The viability of RAW264.7 macrophages was not significantly decreased (*p* > 0.05) by increasing concentrations of GBP, which confirmed that GBP was not cytotoxic to RAW264.7 macrophages (Figure 1B). Moreover, its viability was significantly (*p* < 0.05) lower after the administration of 3.0 μg/mL LPS (51.57 ± 7.07%) than that in CTRL group (100 ± 7.35%). However, with increasing concentrations of GBP pre-treatment, its viability was increased (*p* < 0.05) in the LPS + GBP group. Nevertheless, its viability was not significantly (*p* > 0.05) different between pre-treatment with 200 and 400 μg/mL GBP (Figure 1C). Therefore, the concentrations of LPS (3.0 μg/mL) and GBP (200 μg/mL) were chosen for subsequent experiments. The levels of inflammatory cytokines (IL-1β, IL-6 and TNF-α) are generally considered to be major biomarkers of inflammation [13]. The levels of IL-1β, IL-6 and TNF-α were measured to investigate the effect of GBP on LPS-mediated inflammation in RAW264.7 macrophages. The levels of IL-1β (485.75 ± 50.29 pg/mL), IL-6 (373.01 ± 12.49 pg/mL) and TNF-α (271.35 ± 17.35 pg/mL) were increased (*p* < 0.05) after LPS (1.0 μg/mL) administration compared with those in the CTRL group (Figure 1D–F). However, the levels of IL-1β (353.13 ± 18.12 pg/mL), IL-6 (287.49 ± 8.45 pg/mL) and TNF-α (200.38 ± 9.49 pg/mL) in the LPS + GBP group were decreased (*p* < 0.05), which confirmed that GBP could ameliorate inflammation mediated by LPS in RAW264.7 macrophages.

### 2.2. GBP Was Separated by SEC and Ameliorated LPS Mediated Inflammation in RAW264.7 Macrophages

**Figure 2:**
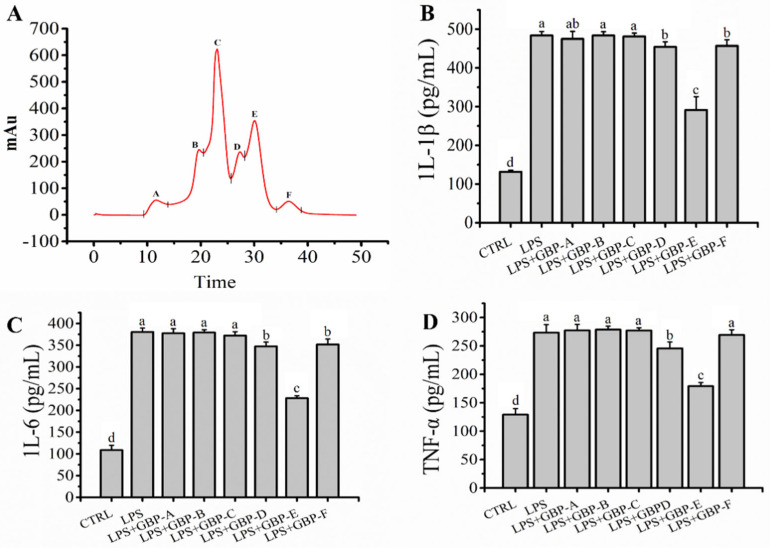
(**A**) GBP was separated to different fractions by SEC; (**B**–**D**): Effects of GBP−(A–F) pre-treatment (200 μg/mL) on levels of IL−1β, IL−6 and TNF−α on model of LPS-Mediated RAW264.7 macrophages damage. Different letters (a, b, c and d) in the figure represent significant differences between groups (*p* < 0.05).

GBP was shown to ameliorate inflammation mediated by LPS in RAW264.7 macrophages as described above. However, GBP is a collection that contains many oligopeptides with molecular weights less than 3 kDa. Identifying which peptides contributed to ameliorating inflammation mediated by LPS in RAW264.7 macrophages is a worthy question for further investigation. In this study, SEC was used to separate the mixture of GBP according to the different molecular masses of the components. As shown in Figure 2A, six fractions, namely A, B, C, D, E, and F, were separated from the mixtures of GBP; fraction A represented the maximum molecular mass and fraction F represented the opposite. The anti-inflammatory activity of the fraction (A–F) obtained by SEC was evaluated in a model of inflammation mediated by LPS in RAW264.7 macrophages in vitro. The levels of IL-1β (291.17 ± 34.53 pg/mL), IL-6 (228.15 ± 5.38 pg/mL) and TNF-α (179.27 ± 6.26 pg/mL) in the fraction E group were significantly lower (*p* < 0.05) than those in the other fraction groups (Figure 2B–D), which confirmed that fraction E showed the strongest anti-inflammatory activity among the fractions. Finally, fraction E was collected and freeze-dried for further analysis.

### 2.3. Fraction E Was Separated by IEC and Ameliorated Inflammation Mediated by LPS in RAW264.7 Macrophages

**Figure 3:**
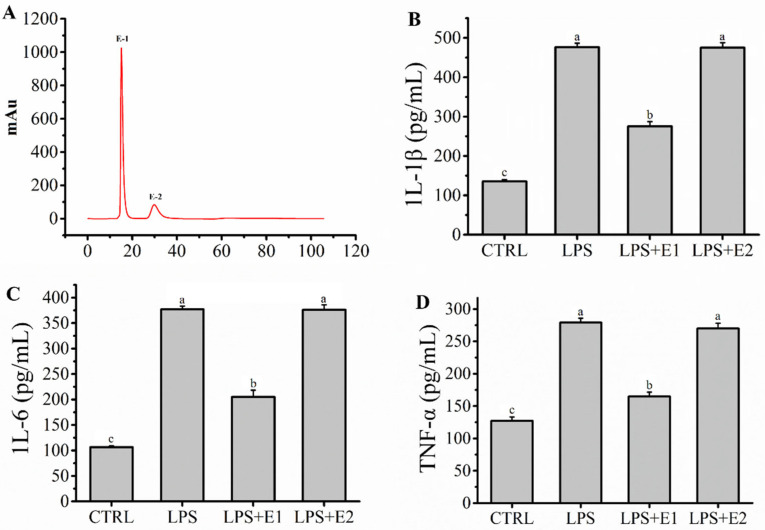
(**A**) HiTrap Capto DEAE ion exchange chromatography of peptides from GBP-E; (**B**–**D**): Effects of GBP-E (1–2) pre-treatment (200 μg/mL) on levels of IL-1β, IL-6 and TNF-α on model of LPS-mediated RAW264.7 macrophages damage. Different letters (a, b and c) in the figure represent significant differences between groups (*p* < 0.05).

Fraction E represented a mixture of peptides with similar small molecular masses. However, these peptide fragments in fraction E may contain groups with different anion exchange capacities. Therefore, fraction E was further separated by IEC depending on the different anion exchange capacities to further elucidate the characteristics of the target peptide. As shown in Figure 3A, the mixture of fraction E was separated into two fractions (E-1 and E-2), and fraction E-1 represented the relatively weaker anion exchange capacity, while fraction E-2 represented the stronger anion exchange capacity. The anti-inflammatory activity of fractions E-1 and E-2 obtained by IEC was evaluated in a model of inflammation mediated by LPS in RAW264.7 macrophages in vitro. Compared to those in the fraction E-2 group, the levels of IL-1β (275.01 ± 12.37 pg/mL), IL-6 (205.14 ± 13.22 pg/mL), and TNF-α (164.76 ± 6.80 pg/mL) in the fraction E-1 group were significantly lower (*p* < 0.05), which confirmed that fraction E-1 showed stronger anti-inflammatory activity than fraction E-2 (Figure 3B–D). Finally, fraction E-1 was collected and lyophilized for further analysis.

### 2.4. Fraction E-1 Was Separated by RP-LC and Ameliorated LPS-Mediated Inflammation in RAW264.7 Macrophages

**Figure 4:**
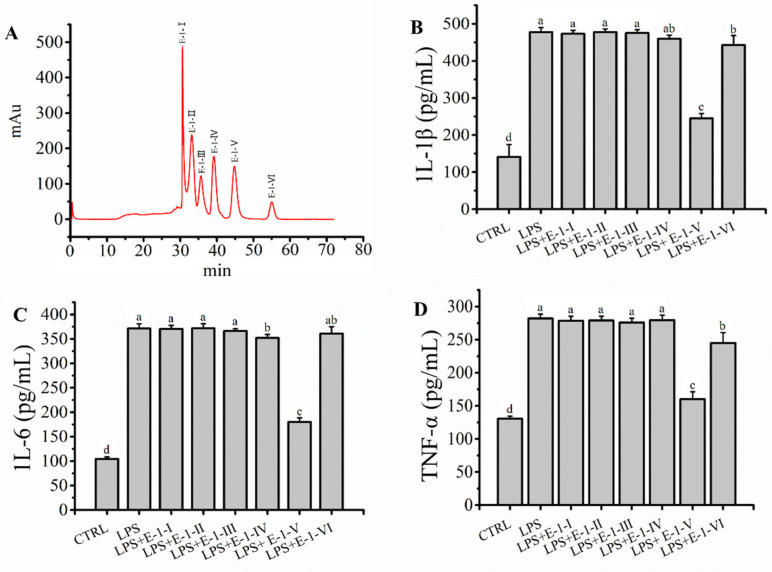
(**A**) BEH C18 reversed-phase liquid chromatography of peptides from E-1; (**B**–**D**): Effects of E-1 (Ⅰ–Ⅵ) pre-treatment (200 μg/mL) on levels of IL-1β, IL-6 and TNF-α on model of LPS-mediated RAW264.7 macrophages damage. Different letters (a, b, c and d) in the figure represent significant differences between groups (*p* < 0.05).

Fraction E-1 represented a mixture of peptides with similar small molecular masses and lower anion exchange capacities. However, these peptide fragments in fraction E-1 may contain groups with differences in hydrophobicity. Therefore, fraction E-1 was further separated by RP-LC depending on differences in hydrophobicity to further elucidate the characteristics of the target peptide. The mixture of fraction E-1 was separated into six fractions (E-1-Ⅰ, E-1-Ⅱ, E-1-Ⅲ, E-1-Ⅳ, E-1-Ⅴ and E-1-Ⅵ) (Figure 4A). The anti-inflammatory activity of fraction E-1-(Ⅰ–Ⅵ) obtained by RP-LC was evaluated in LPS-mediated RAW264.7 macrophages in vitro. As shown in Figure 4B–D, the levels of IL-1β (245.09 ± 12.65 pg/mL), IL-6 (180.14 ± 8.42 pg/mL) and TNF-α (160.04 ± 11.36 pg/mL) in fraction E-1-Ⅴ were greatly lower (*p* < 0.05) than those in the other fractions, which confirmed that fraction E-1-Ⅴ showed the strongest anti-inflammatory activity. Finally, fraction E-1-Ⅴ was collected and freeze-dried for further analysis.

### 2.5. The Target Anti-Inflammatory Peptide Was Determined by LC–MS/MS

**Figure 5:**
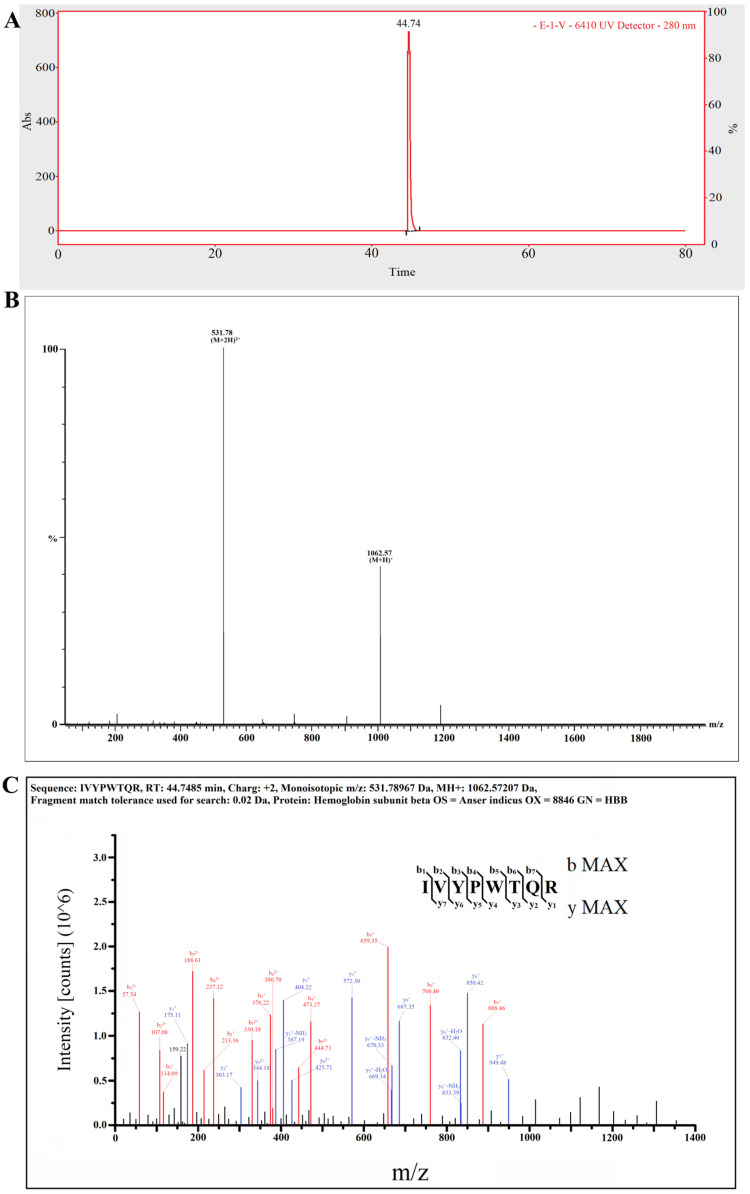
(**A**) The total particles of E−1−Ⅴ in MS/MS spectrum; (**B**) Mass spectrum of peak at 44.74 min; (**C**) Identification of the molecular weight and amino acid sequence of the purified E−1−Ⅴ by MS/MS spectrum.

According to the obtained results, the target GBPs were characterized as having low molecular weights, high hydrophobicity and weak anion exchange capacities. In this study, only one major peak is shown in Figure 5A, which suggested one main target anti-inflammatory peptide in fraction E-1-Ⅴ. As shown in Figure 5B, the molecular ion peak of the target anti-inflammatory peptide was detected at *m*/*z* 531.78 and had a monoisotopic mass of 1062.57207 Da. In addition, the amino acid sequence of the target anti-inflammatory peptide was IIe-Val-Tyr-Pro-Trp-Thr-Gln-Arg (IVYPWTQR), which was derived from haemoglobin subunit beta OS (Figure 5C). The content of IVYPWTQR in the hydrolysates of goose blood was about 2.16%. The characteristics of IVYPWTQR were its low molecular weight, high hydrophobicity (large amount of hydrophobic amino acid residues, such as IIe, Val, Pro and Trp) and weak anion exchange capacity (charge: +2), which conformed to the identified characteristics.

In this study, IVYPWTQR was synthesized by chemical solid-phase synthesis to further verify the anti-inflammatory activity of the target peptide in LPS-mediated RAW264.7 macrophages in vitro. Pre-treatment with IVYPWTQR significantly ameliorated LPS-mediated inflammation in RAW264.7 macrophages in vitro (Figure S1), which further confirmed that IVYPWTQR isolated from enzymatic hydrolysates of goose blood ameliorated LPS-mediated inflammation in RAW264.7 macrophages.

### 2.6. Effects of Pepsin–Trypsin Simulated GI Digestion on the Stability of IVYPWTQR

**Figure 6:**
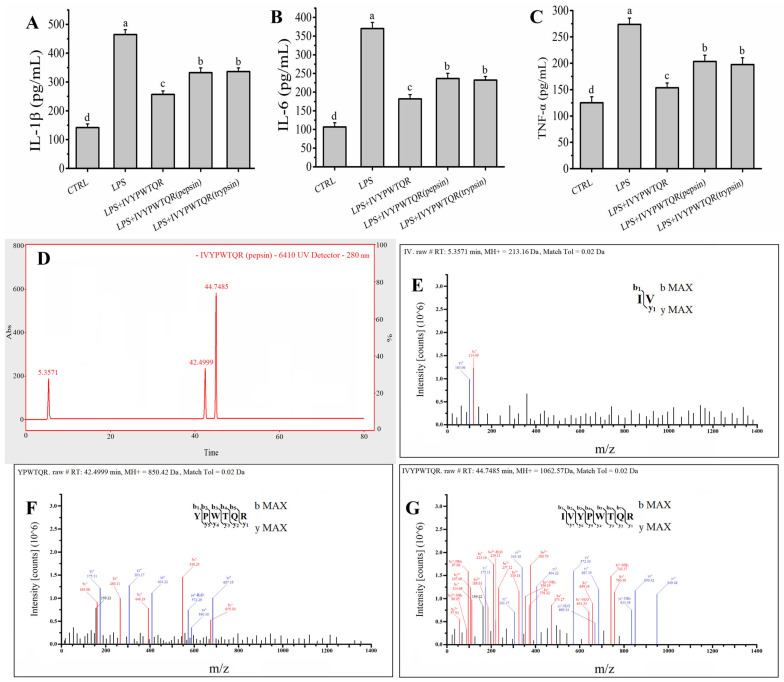
(**A**–**C**) Effects of the IVYPWTQR pre-treatment with pepsin–trypsin-simulated gastrointestinal digestion on the levels of IL−1β, IL−6 and TNF-α on model of LPS-mediated RAW264.7 macrophages damage; (**D**) The total particles of RP-LC of IVYPWTQR pre-treatment with trypsin–simulated intestinal digestion; (**E**–**G**) Identification of the molecular weight and amino acid sequence of peak at 5.3571 min, 42.4999 min and 44.7485 min by MS/MS spectrum; Different letters (a, b, c, d) in figures indicate significant differences (*p* < 0.05).

GI digestion is a major factor that affects the structure and function of bioactive peptides. In this study, the stability of IVYPWTQR during GI digestion was evaluated in a pepsin–trypsin simulated GI digestion model in vitro. The levels of IL-Iβ (332.43 ± 16.46 pg/mL), IL-6 (236.28 ± 13.96 pg/mL) and TNF-α (203.33 ± 12.01 pg/mL) after digestion with pepsin for 2.0 h were slightly increased (*p* < 0.05) compared to the levels of IL-Iβ (256.79 ± 12.04 pg/mL), IL-6 (181.83 ± 11.35 pg/mL) and TNF-α (153.58 ± 8.97 pg/mL) in the LPS + IVYPWTQR group, but these levels were still significantly lower (*p* < 0.05) than the levels of IL-Iβ (464.55 ± 16.97 pg/mL), IL-6 (370.25 ± 16.23 pg/mL) and TNF-α (273.77 ± 11.93 pg/mL) in the LPS group (Figure 6A–C). Furthermore, the levels of IL-Iβ (336.33 ± 12.58 pg/mL), IL-6 (232.34 ± 9.56 pg/mL) and TNF-α (197.44 ± 13.01 pg/mL) after digestion with trypsin for 2.0 h were not significantly increased (*p* > 0.05) compared to the levels of IL-Iβ, IL-6 and TNF-α in the LPS + IVYPWTQR (pepsin) group, indicating that IVYPWTQR robustly resists trypsin (Figure 6A–C). The products of IVYPWTQR digestion with pepsin were analysed by LC–MS/MS. As shown in Figure 6D–F, approximately 45.19% of IVYPWTQR was split into smaller fragments after pepsin treatment; these fragments included IV (20.00%) and YPWTQR (25.19%), indicating that pepsin specifically cleaved the N-terminal peptide bond of the tryptophan residue in IVYPWTQR. This may be why the anti-inflammatory activity of IVYPWTQR decreased after pepsin hydrolysis. This discovery provides insight and a theoretical basis for the design of specific target peptides to avoid enzymatic digestion and reduce peptide hydrolysis. In fact, we are attempting to improve the stability of target bioactive peptides during gastric digestion by lipid embedding.

### 2.7. The Mechanism by Which IVYPWTQR Ameliorated LPS-Mediated Inflammation in RAW264.7 Macrophages

**Figure 7:**
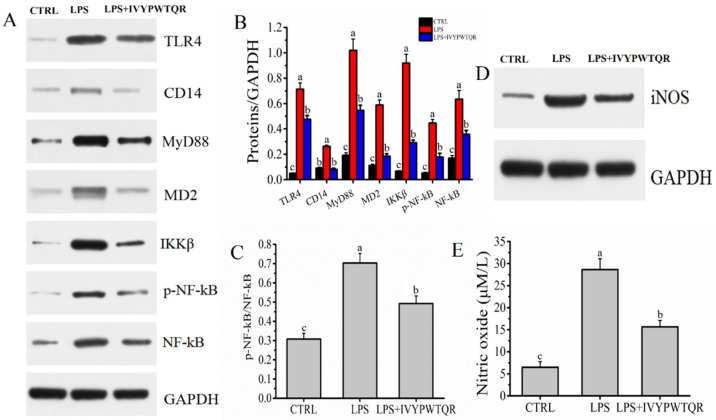
(**A**,**B**) Effects of IVYPWTQR on the levels of LPS receptors (*TLR4*), co-receptors (*CD14* and *MD2*), *MyD88*, *IKKβ*, *p-NF-kB,* and *NF-kB* in LPS-mediated RAW264.7 macrophages; (**C**,**D**) Effects of IVYPWTQR on the levels of *p-NF-kB p65/NF-kB p65* and *iNOS* in LPS-mediated RAW264.7 macrophages; (**E**) Effects of IVYPWTQR on the levels of NO in LPS-mediated RAW264.7 macrophages culture medium. Different letters (a, b and c) in the figure represent significant differences between groups (*p* < 0.05).

LPS is generally considered a critical activator of macrophages and is specifically recognized by receptor proteins on the cell membrane (*TLR4*) and the coreceptors *CD14* and *MD-2*. Subsequently, the adaptor molecule of *MyD88* is recruited to accelerate the phosphorylation of IkB kinase (IKK) to activate *NF-kB* signal transduction pathways, which generates excess proinflammatory cytokines (such as IL-6, TNF-α, and IL-1β) [19,20]. *IKKβ* is a pivotal subunit for activating *NF-kB* signal transduction pathways [21]. In this study, the protein expression levels of the receptor (*TLR4*), coreceptors (*CD14* and *MD-2*), MyD88, *NF-kB*, phosphorylated NF-kB (*p-NF-kB*), and *IKKβ* were measured by Western blot analysis. The protein expression levels for *TLR4*, *CD14*, *MD2*, *MyD88*, *p-NF-kB*, *NF-kB* and *IKKβ* were all substantially (*p* < 0.05) elevated in the LPS group compared to the CTRL group. However, pre-treatment with IVYPWTQR significantly decreased the protein expression levels of *TLR4*, *CD14*, MyD88, *MD-2*, *p-NF-kB/NF-kB,* and *IKKβ* in the LPS + IVYPWTQR group (Figure 7A–C).

TNF-α is generally considered to be a critical proinflammatory mediator of inflammation, which can activate *iNOS* signal transduction pathways to exacerbate inflammation [22]. As shown in Figure 7D, the protein expression level of *iNOS* was significantly (*p* < 0.05) higher in the LPS group than in the CTRL group. However, pre-treatment with IVYPWTQR significantly decreased the protein expression levels of *iNOS* in the LPS + IVYPWTQR group. A previous study had found similar results [23]. The upregulation of *iNOS* signalling promoted the production and accumulation of NO, which was generally considered to be an effector molecule with nonspecific cytotoxicity [24,25]. Compared to that in the CTRL group (6.47 ± 1.29 μM/L), the level of NO was significantly (*p* < 0.05) increased after the administration of LPS (28.63 ± 2.48 μM/L). However, pre-treatment with IVYPWTQR significantly decreased the level of NO (15.65 6.47 ± 1.47 μM/L) in the LPS + IVYPWTQR group (Figure 7E). In summary, IVYPWTQR reduced the production of the proinflammatory cytokines to ameliorate the inflammatory response mediated by LPS by downregulating the expression of the *TLR4/NF-kB/iNOS* signalling pathway.

### 2.8. IVYPWTQR Ameliorated LPS-Mediated Oxidative Stress in RAW264.7 Macrophages

**Figure 8:**
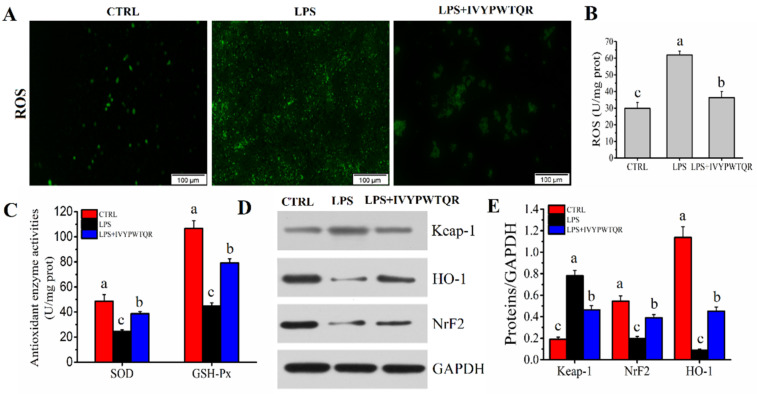
(**A**,**B**) Effects of IVYPWTQR on the levels of ROS in LPS-mediated RAW264.7 macrophages. (**C**) Effects of IVYPWTQR on the levels of antioxidant enzyme activity in LPS-mediated RAW264.7 macrophages. (**D,E**) Effects of IVYPWTQR on the protein expression levels of *Keap-1*, *HO-1*, and *NrF2* in LPS-mediated RAW264.7 macrophages. Different letters (a, b, and c) in the figure represent significant differences between groups (*p* < 0.05).

Macrophages generate excess ROS in response to LPS-mediated inflammation, which accelerates immune dysfunction through oxidative stress [26]. As shown in Figure 8A,B, stimulation with LPS significantly increased the levels of ROS (61.96 ± 2.27 U/mg prot), but the levels of ROS were significantly decreased after pre-treatment with IVYPWTQR (36.32 ± 3.69 U/mg prot). The antioxidant enzymes SOD and GSH-Px are considered key factors for resisting ROS-mediated oxidative stress by scavenging excess ROS in macrophages [27]. Compared to those in the CTRL group, the levels of SOD (24.43 ± 1.41 U/mg prot) and GSH-Px (44.63 ± 2.72 U/mg prot) were significantly (*p* < 0.05) decreased after the administration of LPS. However, pre-treatment with IVYPWTQR significantly increased the levels of SOD (38.68 ± 1.68 U/mg prot) and GSH-Px (79.14 ± 3.41 U/mg prot) in the LPS + IVYPWTQR group (Figure 8C). Therefore, IVYPWTQR could inhibit the production of ROS to ameliorate LPS-mediated inflammation by improving oxidative stress. The *Keap-1/NrF2/HO-1*-mediated pathway is considered a crucial factor in activating and regulating SOD and GSH-Px to participate in antioxidant defence [28,29,30]. As shown in Figure 8D,E, stimulation with LPS significantly increased the protein expression level of *Keap-1, and* decreased the levels of *NrF2* and *HO-1*, but the protein expression levels of *Keap-1* were significantly decreased, and the *NrF2* and *HO-1* were significantly increased after pre-treatment with IVYPWTQR, which confirmed that IVYPWTQR could enhance the antioxidant defence of cells to reduce ROS accumulation and ameliorate the LPS-mediated inflammatory response by activating the *Keap-1/NrF2/HO-1* signalling pathway.

Inflammation is a multifaceted biological process that contributes significantly to many forms of human disease associated with inappropriate diet. Recent evidence indicated that inappropriate diets impaired intestinal barrier function and accelerated the translocation of enterogenous lipopolysaccharide (LPS), which was recognized by TLR4 receptors on macrophages to activate NF-kB pathways and the release of relevant inflammatory factors that lead to inflammation related diseases [31]. Macrophages, especially Kupffer cells, may contribute to the development of inflammation by generating various inflammatory mediators (reactive oxide species (ROS) and nitric oxide) and a series of cytokines (IL-1β, IL-6 and TNF-α) when stimulated by LPS [19]. Furthermore, macrophages generate excess ROS in response to LPS-mediated inflammation, which mediates apoptosis and inflammation, and aggravates disease progression through oxidative stress [26]. In recent years, the use of dietary bioactive compounds to attenuate inflammation and prevent disease aggravation has attracted widespread attention. In this study, an oligopeptide (IVYPWTQR) was isolated and identified from goose blood hydrolysate to ameliorate LPS-induced macrophage inflammation, which had a monoisotopic mass of 1062.5720 Da and was derived from haemoglobin subunit beta OS in goose blood. The content of IVYPWTQR in the hydrolysates of goose blood was about 2.16%. The characteristics of IVYPWTQR were its low molecular weight, high hydrophobicity (large amount of hydrophobic amino acid residues, such as IIe, Val, Pro and Trp) and weak anion exchange capacity (charge: +2). In addition, IVYPWTQR was confirmed to protect against LPS-mediated inflammation response by downregulating the *TLR4/NF-kB/iNOS* pathway and activating the *Keap-1/NrF2/HO-1* signalling pathway to inhibit the production of inflammatory mediators and reactive oxide species (ROS). However, the practical application of target peptides may still face some challenges. Because the target peptide is unstable during gastric digestion, and the absorption of the target peptide in vivo and its interaction with the gut microbiota is still unclear. Of course, this part of the content is the focus of our next study.

## 3. Materials and Methods

### 3.1. Preparation of GBP

Twelve Wanxi white geese (270 days old) were randomly selected and euthanized by CO_2_, and blood was collected from the neck. This study was approved by the animal care and use committee of Anhui Agricultural University (No. SYDW-P20190600601). Goose blood was collected and heated at 95 °C for 5 min to inactivate endogenous enzymes. Then, the goose blood was stirred and freeze-dried to form a powder. One hundred grams of lyophilized goose blood was added to a beaker and then homogenated with 2000 mL PBS (0.01 mol/L, pH = 7.2) in the ice-water bath. GBP preparation was carried out by trypsin enzymatic hydrolysis, which was slightly modified according to the study by Chou et al. [32]. A total of 250 mg of trypsin from porcine pancreas (Purity: ≥250 units/mg, CAS: 9001-75-6, Shanghai Aladdin Biochemical Technology Co., Ltd., Shanghai, China) was accurately weighed and added to the goose blood homogenate to hydrolyse for 4 h at 37 °C. The pH was adjusted to 7.6 with a 0.2 mol/L NaOH solution. Subsequently, the hydrolysate was transferred to 500 mL centrifuge bottles and centrifuged at 12,000× *g* for 20 min at 4 °C to separate the supernatant. The supernatant was filtrated by TFUS with minimate (P/N: S02-E003-05-N, surface area: 790 cm^2^, media/rating: mPES/3 kDa), and the filtrate was collected. Finally, the filtrate was desalted with a C18-SPE column (specification: 500 mg/6 mL). A volume of 6 mL of the filtrate was placed in a C18-SPE column activated by methanol and filtered at a flow rate of 1 mL/min. The sodium chloride in the filtrate was adsorbed in the C18-SPE column, and the filtrate was collected to be freeze-dried for subsequent experiments.

### 3.2. Isolation and Identification of Oligopeptide with Anti-Inflammatory from GBP

The methods for isolation and identification of oligopeptide with anti-inflammatory from the GBP were based on the previous study of Nie et al. [7]. Primarily, the GBP (100 mg/mL) was separated to different fractions depending on different molecular masses by the SEC configured with a Sephadex^TM^ 10/300GL column (General Electric Company, Marlborough, MA, USA). A 0.1 mol/L hydrochloric acid solution was used as mobile phase for separation with flow rate of 1 mL/min. The fractions were detected at 280 nm by an ultraviolet detector and collected by an automatic fraction collector.

The anti-inflammatory activity of the peptides was determined according to the methods of a previous study [13]. The anti-inflammatory activity of different fractions was evaluated by measuring the levels of the inflammatory cytokines (TNF-α, IL-1β and IL-6) in a model of LPS-induced macrophage inflammation with ELISA kits (Jiangsu Enzyme marker Biotechnology Co., Ltd., Yancheng, China). Then, the fraction of peptide with the highest anti-inflammatory activity obtained from SEC was further separated to different fractions depending on different anion exchange capacities. A 100 mg/mL peptide was separated by ion exchange chromatography (IEC) configured with an ion exchange column (HiTrap Capto DEAE, 5 mL, 1.6 × 2.5 cm, General Electric Company, Marlborough, MA, USA). A 20 mmol/L Tris-HCl (pH 8.0) solution was used as the start buffer and 20 mmol/L Tris-HCl/1 mol/L NaCl (pH 8.0) was used as elution buffer. Elution program: (1) wash with 5 column volumes of starting buffer; (2) elute with 10 column volumes of 50% elution buffer and 50% start buffer; (3) elute with 5 column volumes of 100% elution buffer. The above flow rates were 5 mL/min. The fractions were detected at 280 nm by an ultraviolet detector and collected by an automatic fraction collector.

Moreover, the fraction of peptide with the highest anti-inflammatory activity obtained from IEC was further separated to different fractions depending on different hydrophobicity. A 10 mg/mL peptide was separated by the reversed-phase liquid chromatography (RP-LC) with a reverse phase BEH C18 column (1.7 μm, 2.1 × 100 mm, Waters Inc., Milford, MA, USA). Eluent A: 0.065% TFA in 2% acetonitrile; Eluent B: 0.050% TFA in 80% acetonitrile. The gradient elution parameters: 0–30 min, 100% eluent A; 30–60 min, 30–80% eluent B; 60–80 min, 100% eluent A. The above flow rates were 0.5 mL/min. The fractions were detected at 280 nm by an ultraviolet detector and collected by an automatic fraction collector.

The fractionation of the peak with the highest anti-inflammatory activity obtained from RP-LC was further identified by LC-MS/MS equipped with a reversed phase BEH C18 analytical column (1.7 μm, 2.1 × 100 mm, Waters Inc., Milford, MA, USA). The fraction of peptide with the highest anti-inflammatory activity obtained from RP-LC was further Identified by LC-MS/MS. Eluent A: 0.065% TFA in 2% acetonitrile; Eluent B: 0.050% TFA in 80% acetonitrile. The gradient elution parameters: 0–30 min, 100% eluent A; 30–60 min, 30–80% eluent B; 60–80 min, 100% eluent A. The above flow rates were 0.5 mL/min. The fractions were detected at 280 nm by an ultraviolet detector. The flow entered directly into the MS/MS system (MALDI SYNAPT Q-TOF MS, Waters, Inc. Milford, MA, USA) for multiple reaction measurement. The recording mass range of precursor ions was *m*/*z* 50–4000. Nitrogen was used as a collision gas. Capillary voltage was set at 3.5 kV and cone voltage at 20 V. Source block temperature was set at 100 °C and desolvation temperature at 250 °C. Collision energy and detector voltage were 15 and 1600 V, respectively. The desolvation and cone gas flow were 500 and 50 L/h, respectively. The N-terminal fragment ion series (B series) and the C-terminal fragment ion series (Y series) were analysed. The amino acid sequence of the peptide was obtained by comprehensive analysis of B and Y ion series through Mass Lynx V4.1 operating system.

### 3.3. Cell Culture

The method of RAW264.7 macrophage culture and treatments were based on the study of Li et al. [13]. RAW264.7 macrophages (Lot number: BH0799, Chinese Academy of Sciences, Shanghai, China) were grown in DMEM-F medium (1:1) containing 3.15 g/L glucose, 0.365 g/L l-glutamine, 3.57 g/L HEPES, 10% FBS and 1% antibiotics at 37 °C in an atmosphere with 5% CO_2_ and 90% relative humidity. Macrophages from passages 5 to 10 were selected to seed on 96-well culture plates at a density of 4 × 10^5^ cells/well and cultured with different concentrations of LPS (0.5 μg/mL, 1.0 μg/mL and 2.0 μg/mL) for 24 h. The optimal concentration of LPS for inducing inflammation in the RAW264.7 macrophage was ensured according to cell viability. Furthermore, RAW264.7 macrophages were cultured with different concentrations of GBP (100 μg/mL, 200 μg/mL and 400 μg/mL) to evaluate the cytotoxicity of GBP as described above. The ability of GBP to protect RAW264.7 macrophages was evaluated. In the LPS + GBP group, the macrophages medium supplemented with 200 μg/mL GBP was incubated for 2 h. Then, the macrophages medium was substituted with a macrophages medium supplemented with 1.0 μg/mL LPS and incubated for 24 h. In the LPS group, standard macrophages medium incubated for 2 h. Then, the standard macrophages medium was substituted with macrophages medium supplemented with 1.0 μg/mL LPS and incubated for 24 h. In the CTRL group, standard macrophages medium incubated for 2 h, Then, the standard macrophages medium was replaced with a new one and incubated for 24 h. Finally, the macrophages medium and macrophages were collected for further analysis.

### 3.4. Simulated Gastrointestinal (GI) Digestion

The stability of GBP in response to pepsin and trypsin in the simulation of GI digestion in vitro was examined based on Xie et al. [33]. First, 2 g of NaCl and 3.2 g of pepsin from porcine gastric mucosa (Purity: 1:3000, CAS: 9001-75-6, Shanghai Aladdin Biochemical Technology Co., Ltd., Shanghai, China) were added to 800 mL of ultrapure water. The pH of the solution was adjusted with 6 mol/L hydrochloric acid to 3.0, and the volume was adjusted with ultrapure water to 1 L (as simulated gastric juice). Then, 0.68 g of potassium dihydrogen phosphate and 7.7 mL sodium hydroxide solution (0.2 mol/L) were dissolved in 70 mL of ultrapure water; 1 g of trypsin from porcine pancreas (Purity: ≥250 units/mg, CAS: 9001-75-6, Shanghai Aladdin Biochemical Technology Co., Ltd., Shanghai, China) and 6 g of bile salt were added and dissolved. The above solution was mixed and supplemented with ultrapure water to 1 L. Finally, the pH of the mixed solution was adjusted to 7.6 with sodium hydroxide solution (0.2 mol/L) (as simulated intestinal fluid). The 200 μg/mL peptide solution was adjusted to a pH of 3.0 with a 1 mol/L HCl solution, and 5 mL of simulated gastric juice was added to the solution and the reaction was carried out for 2 h in 37 °C water bath. Then, the simulated gastric digestion was terminated by being heated at 100 °C for 10 min. The pH of simulated gastric digestive solution was modulated to 7.6 with sodium hydroxide solution (0.2 mol/L). A volume of 5 mL of simulated intestinal juice was added to the solution, and the mixture was shaken in 37 °C water bath for 2 h. Finally, the digestive liquid was transferred to the ultrafiltration centrifuge tube (2 kDa with centrifuging at 8500× *g*, 4 °C for 20 min). Finally, the filtered solutions were collected and desalted by a polar enhanced polymer (PEP) column (500 mg/6 mL).

### 3.5. Determination of Cytokines and Oxidative Stress Indicators

The culture medium was collected and centrifuged at 3000× *g* for 10 min. Then, the inflammatory cytokines levels of TNF-α (MB-50051A), IL-1β (MB-50064B) and IL-6 (MB-50054A) in the culture medium were determined with ELISA kits (Jiangsu Enzyme marker Biotechnology Co., Ltd., Yancheng, China). Steps: (1) Remove the required lath from the aluminium foil bag after 20 min equilibration at room temperature; (2) Set standard wells and sample wells, and add different concentrations of standard 50 μL to standard wells; (3) Add 10 μL of sample to the sample well, and then add 40 μL of sample dilution (blank well without sample); (4) In addition to the blank wells, 100 μL horseradish peroxidase labelled antibody was added to each well of the standard and sample wells, and the reaction wells were sealed with a sealing plate membrane and incubated at 37 °C for 60 min. (5) Discard the liquid and fill each well with the washing liquid, stand for 1 min, discard the washing liquid, and repeat washing the plate for 5 times. (6) Add 50 μL of substrate A and B were to each well and incubate at 37 °C for 15 min. (7) Add 50 μL of stop solution to each well, and measure its absorbance value at 450 nm wavelength. The activities of SOD, GSH-Px and the levels of ROS and Nitric oxide (NO) in macrophages were determined with spectrophotometric kits. SOD: 20 μL 0 f sample was mixed with 20 μL enzyme working liquid and 200 μL substrate application solution. Then miscible liquids were performed at 37 °C for 20 min. The absorbance value of miscible liquid was measured at 450 nm wavelength by a multifunctional microplate reader; GSH-Px: 0.2 mL of sample was mixed with 0.2 mL GSH-Px (1 mmol/L) and performed at 37 °C for 5 min. Then 0.1 mL reagent one was added and performed at 37 °C for 5 min. Subsequently, the miscible liquids were centrifuged at 4 °C, 3500× *g* for 10 min. A volume of 1 mL of supernatant was mixed with 1 mL reagent three, 0.25 mL reagent four, and 0.05 mL reagent five, and performed at room temperature for 15 min. The absorbance value was measured at 412 nm wavelength by a multifunctional microplate reader; ROS: DCFH-DA probe was added directly to serum-free medium: DCFH-DA was generally diluted with serum-free medium at a final concentration of 10 µM at 1:1000. After removing the culture medium, an appropriate volume of diluted DCFH-DA was added. The volume added should be sufficient to cover the cells. Usually, no less than 1 mL of diluted DCFH-DA was added to one well of a 6-well plate. A sample of cells without probe and medium only was set as negative for control. Positive control: Take a sample of cells with probe, and add reactive oxygen species to induce cells. Cells were incubated at 37 °C for 20 min (mixed upside down every 3 to 5 min to make full contact between probe and cell). The length of incubation time was related to cell type, stimulation condition, and DCFH-DA concentration. In general, obvious green fluorescence can be observed in positive control cells after 20 min of stimulation. The culture medium was aspirated, and the serum-free culture medium or 0.01 MPBS was used to blow repeatedly. The bottom of the bottle was observed to be transparent from translucent (cell monolayer connected into slices), and almost all the cell layer was blown into PBS. The cell suspension was completely collected into a 1.5 mL centrifuge tube. The DCFH-DA was washed twice with serum-free medium or PBS to fully remove the DCFH-DA that did not enter the cells. At 1000 rpm/min for 5 min, the supernatant was aspirated and then the cells were resuspended with PBS for determination. Wavelength setting: the optimal excitation wave-length is 488 nm, and the optimal emission wavelength is 525 nm. It can also be detected according to FITC fluorescence detection conditions. The results were expressed as fluorescence values.

### 3.6. Western Blot Analysis

RAW264.7 macrophages were washed with cold PBS and homogenized with two small magnetic beads (2 mm) and 10× volume of RIPA buffer containing 1% protease inhibitors for 60 s. Then the supernatant was collected after centrifuging at 12,000× *g* for 10 min at 4 °C, which was the total protein solution. The concentration of protein in the total protein solution was measured by BCA kits. The protein concentration of the supernatant was diluted to 2.0 mg/mL and mixed with an equal volume of buffer (0.25 mol/L Tris-HCl buffer, pH 6.8, containing 4% SDS and 20% glycerol with 20 mmol/L 2-mercaptoethanol). The stacking gel and separating gel were made of 5% (*w*/*v*) and 10% (*w*/*v*) polyacrylamide, respectively. Electrophoresis was carried out at 80 V until the dye front reached the bottom of the stacking gel, and the gel was run at 120 V until the dye front reached the bottom of the separating gel (165-8001, Bio-Rad Mini-PROTEAN Tetra Electrophoresis System). The PVDF membrane was covered on the gel and transferred membrane at 300 mA for 30 min. The membrane was moved into a decolorization shaker with 5% skim milk (0.5% TBST) and blocked for 1 h at room temperature. The primary antibody was diluted according to the antibody instructions (ab217274, TLR4, 1:1000; ab174606, MD2, 1:1000; ab76302, p-NF-kB, 1:1000, ab32536, NF-kB, 1:3000; ab221678, CD14, 1:1000; ab124957, IKKβ, 1:3000; ab178945, iNOS, 1:1000; ab92946, NRF2, 1:1000; ab219413, MyD88, 1:3000; ab305290, HO-1, 1:1000; ab227828, Keap-1, 1:3000). Then, the blocking solution in the incubation tank was poured out, the configured primary antibody was added, and incubated in the shaker at 4 °C overnight. The secondary antibody (ab105113) was diluted 3000 times with TBST and incubated for 30 min at room temperature. All antibodies were purchased from Abcam Trading Co., Ltd. (Shanghai, China). The secondary antibody was washed three times with TBST on the decolorization shaker for 5 min each time. Finally, the mixed ECL solution was added for full reaction, and the exposure was started after 2 min. The exposed film is developed and fixed with developing and fixing reagents. The film was scanned and archived, processed and decolorized by Photoshop, and the optical density value of the target band was analysed by ImageJ software processing system.

### 3.7. Statistical Analysis

All the results were repeated three time and expressed as mean ± S.D. Comparisons across groups were measured using ANOVA and then Duncan’s multiple comparison test at *p* < 0.05.

## 4. Conclusions

In conclusion, the amino acid sequence of the target anti-inflammatory active peptide was IIe-Val-Tyr-Pro-Trp-Thr-Gln-Arg (IVYPWTQR), which had a monoisotopic mass of 1062.5720 Da and was derived from haemoglobin subunit beta OS in goose blood. However, IVYPWTQR is sensitive to pepsin. Therefore, we then attempted to overcome the degradation of IVYPWTQR during gastric digestion by means of lipid embedding. The mechanism by which IVYPWTQR protects against LPS-induced inflammation was attributed to the fact that IVYPWTQR downregulated the expression of the *TLR4/NF-KB/iNOS* signalling pathway to inhibit the inflammatory cascade and activated the *Keap-1/NRF2/HO-1* antioxidant defence system to resist ROS-mediated oxidative stress. In summary, this study provided a new possibility for high-value processing and utilization of goose blood by-products. Simultaneously, it provided an economical and feasible strategy to prevent or mitigate LPS-mediated inflammation caused by inappropriate diet through dietary supplement of functional factors.

## Data Availability

The original contributions presented in this study are included in the article/Supplementary Materials, further inquiries can be directed to the corresponding authors.

## Supplementary Materials

The following supporting information can be downloaded at: https://www.mdpi.com/article/10.3390/molecules27248816/s1, Figure S1: (A) The total particles of solid phase synthesis of IVYPWTQR in MS/MS spectrum; (B) Mass spectrum of peak at 44.7485 min; (C) Identification of the molecular weight and amino acid sequence of the purified IVYPWTQR by MS/MS spectrum; (D-F): Effects of IVYPWTQR pre-treatment on levels of IL-1β, IL-6 and TNF-α on model of LPS-induced RAW264.7 macrophages damage. Samples designated with different lower cases letters (a, b, c) were significantly different (*p* < 0.05) when compared different treatment group. Table S1: Feed composition and nutritional concentration.
