# Supplementary material for: Isolation and Identification of Anti-Inflammatory Peptide from Goose Blood Hydrolysate to Ameliorate LPS-Mediated Inflammation and Oxidative Stress in RAW264.7 Macrophages
Source: Molecules. 2022 Dec 12;27(24):8816. doi: 10.3390/molecules27248816 (PMC9781827; doi:10.3390/molecules27248816)
Supplement: Supplementary file 1 [file molecules-27-08816-s001.zip › molecules-1997756-supplementary.pdf]

molecules

Supplementary Information

Isolation and Identification of Anti-Inflammatory Peptide from Goose Blood Hydrolysate to Ameliorate LPS-Mediated Inflammation and Oxidative Stress in RAW264.7 Macrophages

Yeye Du [1,2], Shuangjie Zhu [2], Ran Wang [1], Xingyong Chen [3,*] and Kezhou Cai [1,*]

1 Engineering Research Center of Bio-Process, Hefei University of Technology, Ministry of Education, Hefei 230009, China
2 School of Biological and Food Engineering, Chuzhou University, Chuzhou 239001, China
3 College of Animal Science and Technology, Anhui Agricultural University, Hefei 230036, China
* Correspondence: chenxingyong@ahau.edu.cn (X.C.); kzcai@hfut.edu.cn (K.C.); Tel.: +86-551-65785519 (X.C.); +86-15305516697 (K.C.)

Citation: Du, Y.; Zhu, S.; Wang, R.; Chen, X.; Cai, K. Isolation and Identification of Anti-Inflammatory Peptide from Goose Blood Hydrolysate to Ameliorate LPS-Mediated Inflammation and Oxidative Stress in RAW264.7 Macrophages. *Molecules* **2022**, *27*, 8816. https://doi.org/10.3390/molecules27248816

Academic Editor: Warren McNabb

Received: 13 October 2022
Accepted: 6 December 2022
Published: 12 December 2022

Publisher's Note: MDPI stays neutral with regard to jurisdictional claims in published maps and institutional affiliations.

Copyright: © 2022 by the authors. Submitted for possible open access publication under the terms and conditions of the Creative Commons Attribution (CC BY) license (https://creativecommons.org/licenses/by/4.0/).

Table S1. Feed composition and nutritional concentration.

Ingredients	%	indicators	Nutrient concentration
Corn	63	Metabolic energy (MJ/kg)	11.24
Soybean meal	20	Crude Protein (%)	15.14
Wheat bran	10.5	Lysine (%)	0.76
Line powder	1.5	Methione (%)	0.26
Premix	5	Ca (%)	1.63
-	-	P (%)	0.69

Solid-phase synthesis and validation of IVYPWTQR

(1) Accurately weigh 0.2mmol chlorine resin into the peptide synthesis tube, add DMF and DCM at 1:1, stand for 30min, make the resin fully swelling;

(2) according to the ratio of amino acid: shrinkage agent: DIEA: resin = 4:4:8:1, add weighing and placed in 10mL centrifuge tube for use;

(3) Dissolve the first amino acid with 3mL DMF (excluding shrinkage mixture), fully dissolve and add to the polypeptide synthesis tube equipped with resin, placed in the air bath shaker, under the condition of 35°C, 140rmp shock 8h;

(4) The liquid in the polypeptide synthesis tube was drained, and the resin was cleaned 3 times with DMF, 3 times with DCM, and 3 times with DMF;

(5) Add 4mL of 5% methanol solution to the air bath shaker and shake for 30min to completely seal the unreacted resin;

(6) Repeat the operation of step (4);

(7) Mix piperidine and DMF 1:4, configure into 20% piperidine solution, add 5mL of 20% piperidine solution to the peptide synthesis tube, and put it in the air bath shaker, under the condition of 35°C, 140rmp shock for 15min;

(8) Connect the second amino acid, the beforehand weighed good amino acid and shrinkage mixture with 4mL DMF dissolved, and then poured into the peptide synthesis tube, and put it in the air bath shaker, under the condition of 35°C, 140rmp shock for 30min. Repeat steps (6), (7), (8) until all the amino acids have been linked;

(9) Repeat the operation of step (4);

(10) TFA, phenol, deionized water and TIPS were mixed at the ratio of 88:5:5:2 to form a cutting solution, and then 5mL of cutting solution was added to the peptide synthesis tube, which was placed in an air bath shaker and shaken for 3h at 35°C and 140rmp;

(11) Press out all the liquid in the polypeptide synthesis tube and concentrate it to about 1mL by nitrogen blowing instrument;

(12) Add 5mL of iced diethyl ether to precipitate the polypeptide in the solution;

(13) After centrifugation at 4°C and 4000rmp for 10min, the upper solution was discarded and the precipitate was taken for drying, which was the target peptide.

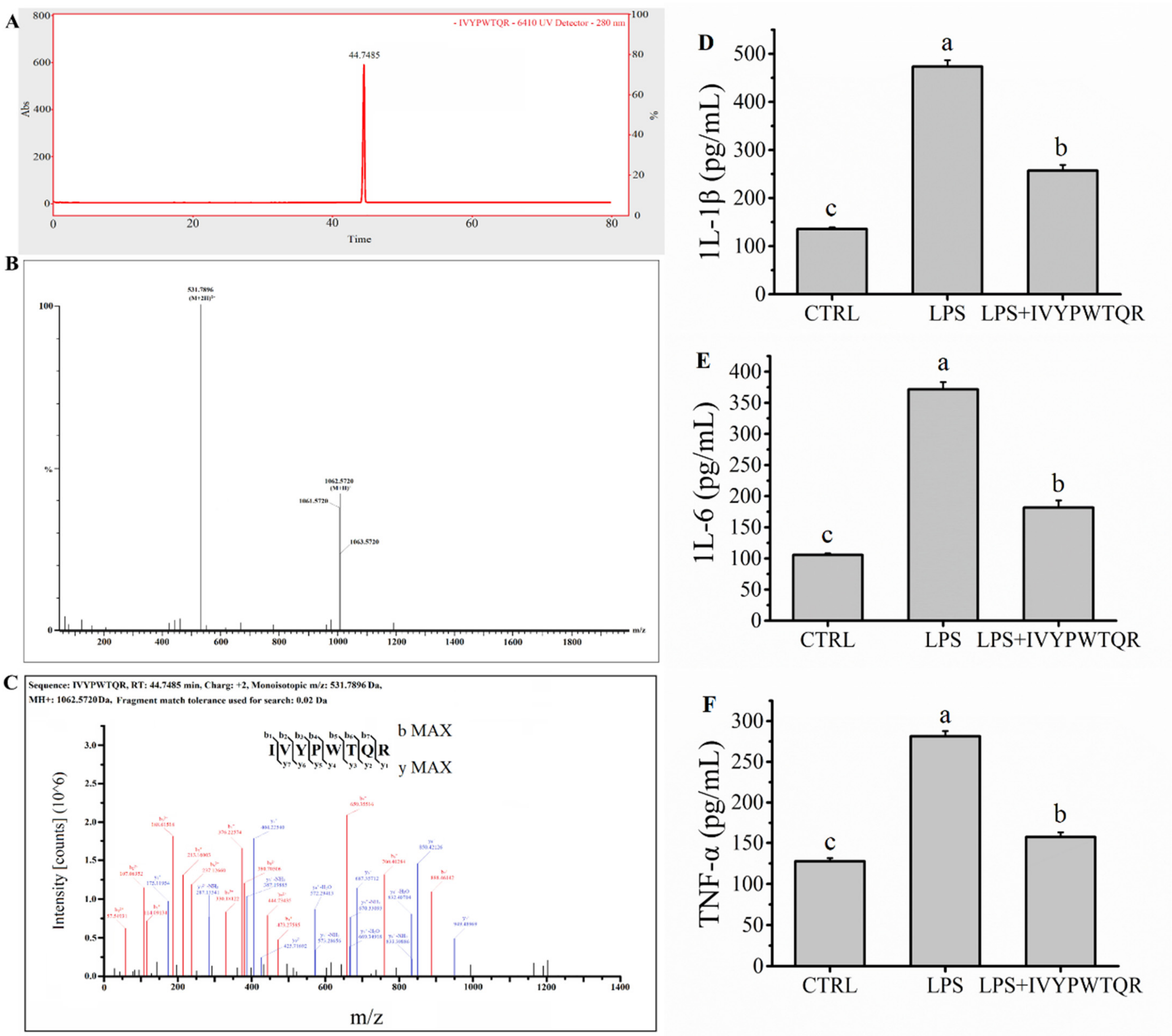

Figure S1. (A) The total particles of solid phase synthesis of IVYPWTQR in MS/MS spectrum; (B) Mass spectrum of peak at 44.7485 min; (C) Identification of the molecular weight and amino acid sequence of the purified IVYPWTQR by MS/MS spectrum; (D-F): Effects of IVYPWTQR pre-treatment on levels of IL-1β, IL-6 and TNF-α on model of LPS-induced RAW264.7 macrophages damage. Samples designated with different lower cases letters (a, b, c) were significantly different ($P < 0.05$) when compared different treatment group.
